# Causal Graphical Model of Bacterial Vaginosis in Pregnant Women

**DOI:** 10.3390/diseases13110375

**Published:** 2025-11-15

**Authors:** Maricela García-Avalos, Juana Canul-Reich, Lil María Xibai Rodríguez-Henríquez, Erick Natividad De la Cruz-Hernández

**Affiliations:** 1 Academic Division of Science and Information Technology, Universidad Juárez Autónoma de Tabasco, Cunduacan-Jalpa de Mendez Road Km. 1, Cunduacán 86690, Tabasco, Mexico; maricela.garcia@ujat.mx; 2 Coordination of Computational Sciences, National Institute of Astrophysics, Optics, and Electronics, Luis Enrique Erro No. 1, Tonantzintla, San Andrés Cholula 72840, Puebla, Mexico; lmrodriguez@inaoep.mx; 3 SECIHTI, Av. de los Insurgentes Sur 1582, Crédito Constructor, Benito Juárez, Ciudad de Mexico 03940, Mexico; 4 Multidisciplinary Academic Division of Comalcalco, Universidad Juárez Autónoma de Tabasco, Rancheria Sur 4th Section, Comalcalco 86658, Tabasco, Mexico; erick.delacruz@ujat.mx

**Keywords:** path analysis, correlation matrix, variability, statistical metrics

## Abstract

Background: This study developed a Causal Graphical Model (CGM) to analyze Bacterial Vaginosis (BV), a condition caused by an imbalance in the vaginal microbiota, whose bacterial composition varies among women. While previous studies used variable selection, clustering, and association rules to identify BV-associated bacteria, these approaches lack visual tools to explore causal relationships and determine which are the most relevant. In contrast, the CGM generated in this study allows visualization of associated bacteria and their causal links, thereby identifying those most influential. Methods: Path Analysis (PA), a statistical structural equation modeling method, was used to construct the CGM, with emphasis on observable variables and to assess direct and indirect effects through correlations and covariances. PA was applied to an already-collected third-party dataset related to BV diagnosis, consisting of data from 132 pregnant women between 4 and 24 weeks of gestation. Results: The CGM, built using a theoretical model based on the Spearman correlation matrix, was validated through statistical metrics and by a clinical-biological expert. The resultant model highlights bacteria influencing BV diagnosis, specifically *Mycoplasma hominis* (Mh), *Atopobium vaginae* (Av), *Gardnerella vaginalis* (Gv), *Megasphaera Type 1* (MT1), and *Bacteria Associated with Bacterial Vaginosis Type 2* (BVAB2). Among them, MT1 and BVAB2 showed the strongest association with BV. Conclusions: The CGM effectively identifies causal associations among bacteria related to BV.

## 1. Introduction

Bacterial Vaginosis (BV) is a common microbiological disorder in women of reproductive age, affecting over 30% of the population, with symptoms occurring in approximately half of the cases [1,2]. When symptomatic, BV manifests as a homogeneous, foul-smelling vaginal discharge, with a distinctive fishy odor that intensifies after sexual intercourse [3,4].

In addition to the impact on women’s quality of life, BV is associated with an imbalance in the vaginal microbiota, which can lead to gynecological and obstetric complications [3]. BV is not caused by a single pathogen but rather by a consortium of anaerobic bacteria, including *Gardnerella vaginalis*, *Atopobium vaginae*, *Bacteria associated with bacterial vaginosis Type 2*, *Megasphaera Types 1 and 2*, whose roles vary across populations and clinical contexts [4]. Moreover, recent studies highlight that BV may follow multiple etiological pathways depending on host–microbe interactions, and that microbial profiles differ significantly across geographic and ethnic populations [2,5]. This study aims to identify coexisting bacterial species influencing BV diagnosis in pregnant women.

Machine learning techniques, including attribute selection, clustering, and association rules, have been used to identify bacteria associated with BV [6,7,8]. These approaches help analyze large datasets, improving diagnostic efficiency and treatment strategies [9]. However, while these methods identify relevant associations, they lack visual tools to represent causal relationships and determine which bacteria are most influential. Incorporating such representations would support healthcare professionals in making more accurate diagnoses and defining appropriate treatments for pregnant women with BV.

Recently, a study applied Bayesian Networks (BNs) to a BV dataset, generating a graphical model that, like Path Analysis (PA), represents the relationships between variables. However, while BNs show probabilistic dependencies between bacterial species and clinical factors [10], PA quantifies the strength and direction of direct and indirect effects through path coefficients, providing the total causal effect between bacteria and the diagnosis. Therefore, Path Analysis provides a more informative and suitable framework for causal inference in this study.

Path Analysis (PA) is a multivariate statistical method within structural equation modeling that examines direct and indirect effects among observable variables, using correlations and covariances [11,12,13]. In this study, PA was applied using R software [14] on BV diagnosis data from pregnant women in Tabasco, Mexico. The resulting Causal Graphical Model (CGM) identified five bacteria influencing BV diagnosis: *Mycoplasma hominis* (Mh), *Atopobium vaginae* (Av), *Gardnerella vaginalis* (Gv), *Megasphaera Type 1* (MT1), and *Bacteria Associated with Bacterial Vaginosis Type 2* (BVAB2). The first four bacteria align with previous findings from machine learning models [6,7,8].

Unlike previous techniques, our CGM provides a deeper causal analysis, revealing that MT1 and BVAB2 are the most influential bacteria in BV diagnosis with total effect values of 0.4900 and 0.5750, respectively. In contrast, the values of Mh (−0.1937), Av (0.2627), and Gv (0.0482) reflect differentiated contributions: Mh presents an inverse association, Av a moderate influence, and Gv a low participation.

This study is described in the rest of the paper as follows: Section 2 presents the materials and methods used. Section 3 describes the experimental study, data preprocessing, and the application of Path Analysis to construct the Causal Graphical Model. Section 4 reports the results with focus on the most influential bacteria in the diagnosis of Bacterial Vaginosis. Section 5 discusses the findings with respect to previous studies. Finally, Section 6 outlines the study’s conclusions.

## 2. Materials and Methods

### 2.1. Spearman Correlation Matrix

Correlation determines the relationship between two quantitative variables, assessing both direction and intensity. Covariance measures how two random variables vary together, and through standardization, it gives out the correlation coefficients [15].

These coefficients range from −1 to 1, where 0 indicates no correlation. A positive value represents a direct relationship, while a negative value denotes an inverse correlation [15].

The Spearman correlation coefficient [16,17], unlike Pearson’s, does not require normality or a strictly linear relationship. It is computed using Equation (1).

(1) $$\rho_{s}=1-\frac{6\sum d_{i}^{2}}{n(n^{2}-1)}$$

where

- $\rho_{s}$: Spearman correlation coefficient.
- $d_{i}$: Difference between ranks of observation pairs.
- *n*: Total number of observations.

### 2.2. Path Analysis

Structural Equation Models (SEM) are multivariate statistical tools, also known as covariance structure analysis, that examine direct (causal) and indirect relationships between variables. SEM incorporates both observable and latent variables, with Path Analysis (PA) being a method specifically focused on observable variables, identifying direct and indirect effects among them [18,19].

**Figure 1:**
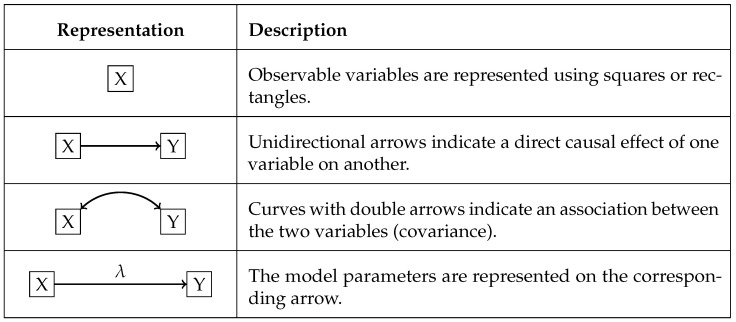
Graphical representation of the PA model, illustrating the causal relationships among variables through observable effects and associations, adapted from [13,19].

The graphical conventions of the PA model [13,19] are illustrated in Figure 1, which visually represents causal relationships among variables through observable associations.

In PA, variables are categorized into two types [19,20]:

- Endogenous: Affected by other variables within the model.
- Exogenous: Not influenced by other variables in the same context.

Types of effects in a path diagram, classified according to their influence on variable interactions [13,19]:

- Direct effect: Influence of one variable on another without intermediaries.
- Indirect effect: A mediating variable connects independent and dependent variables.
- Total effect: Sum of direct and indirect effects.

### 2.3. Statistical Metrics in Path Analysis

Statistical metrics assess model performance relative to observed data by quantifying its fit and predictive capacity. Table 1 presents metrics used in PA [18,21] to evaluate whether the theoretical model adequately represents the underlying data structure.

RMSEA and SRMR are absolute fit indices that evaluate how well the covariance matrix aligns with the implicit model, where lower values indicate better fit quality. GFI, NFI, and CFI are incremental fit indices that measure improvements over a baseline model [21,22].

### 2.4. Kolmogorov–Smirnov Test

In statistics, parametric and non-parametric tests are widely used for data analysis. One nonparametric test is the Kolmogorov–Smirnov test, which assesses whether a sample follows a normal distribution.

This test considers two hypotheses at a significance level of p≥0.05:

- $H_{0}$: The data follow a normal distribution.
- $H_{1}$: The data do not follow a normal distribution.

The Kolmogorov–Smirnov statistic, presented in Equation (2), measures the maximum vertical distance between empirical cumulative distribution functions or between an empirical and a theoretical cumulative distribution function [23,24]. The test determines whether the null hypothesis should be rejected based on the significance level.

(2) $$D_{n}=\underset{x}{\sup}\left|F_{n}\left(x\right)-F\left(x\right)\right|$$

where

- $D_{n}$: Kolmogorov–Smirnov statistic, which measures the maximum distance.
- $\sup_{x}$: Supremum (the maximum value) of the absolute differences between the functions.
- $F_{n}\left(x\right)$: Empirical cumulative distribution function based on the sample data.
- F(x): Theoretical cumulative distribution function under the null hypothesis.

### 2.5. Q-Q Plots

Normal quantile plots, or Q-Q plots, compare a dataset’s distribution to a theoretical distribution, such as the normal distribution [23]. In these plots, observed data quantiles are on the horizontal axis, while expected quantiles from the theoretical distribution are on the vertical axis. When the data follow the theoretical distribution, the points align diagonally. Deviations indicate potential mismatches between the dataset and the theoretical model.

### 2.6. Data Transformation

Transformations are applied when data do not follow a normal distribution, in order to improve the stability and interpretability of the models. The transformations applied in this study are as follows [25,26]:

- Logarithmic transformation: Used for right-skewed positive data; a constant can be added if zero or negative values are present. It is defined as Equation (3).
  (3) y=log(x+a).
- Square root transformation: Suitable for non-negative count data with moderate right skewness, especially when the variance increases with the mean, and represented as Equation (4).
  (4) $$y=\sqrt{x}.$$
- Reciprocal transformation: Helps reduce the impact of large values but cannot be applied to zero, as the reciprocal is undefined. It is given by Equation (5).
  (5) $$y=\frac{1}{x}.$$
- Box-Cox transformation: A generalized approach requiring positive data, expressed in Equation (6).

(6) $$y=\left\{\begin{array}{cc} \frac{x^{\lambda }-1}{\lambda }, & \mathrm{si}\;\lambda \neq 0.\\ \log(x), & \mathrm{si}\;\lambda =0. \end{array}\right.$$

where *x* denotes the original value, *y* the transformed value, *a* shift constant and λ a parameter estimated from the data to enhance normality.

## 3. Experimental Study

The study was conducted in two phases: the first involved preprocessing the dataset, and the second consisted of implementing path analysis to build the causal graphical model. All analyses were performed in R version 4.3.2 (Windows 11), using the following functions and packages:

- Statistical functions.
- Lavaan for SEM/PA.
- SemPlot for plotting.

The dataset and R scripts are available upon request.

### 3.1. Data Preprocessing

Data preprocessing is essential for any statistical analysis or machine learning model, as it includes steps such as imputation of missing values, handling of outliers, removal of irrelevant variables, among others. These procedures enhance dataset quality, reduce bias, and ensure more reliable and reproducible results [27,28]. It is important to note that the dataset does not exclusively contain variables related to bacterial vaginosis, as it was designed to support multiple types of studies.

**Figure 2:**
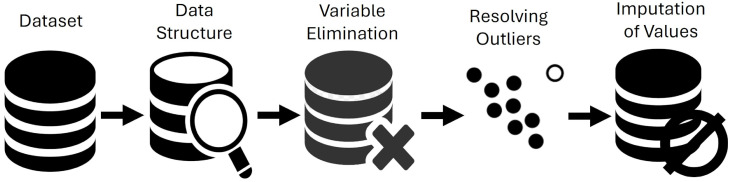
Stages applied during the development of the analysis.

As shown in Figure 2, data preprocessing involved five steps: (a) understanding the source of the dataset, (b) analyzing its structure, (c) removing irrelevant variables, (d) addressing outliers, and (e) imputing missing values to ensure consistency and analytical readiness.

(a) Dataset: Data were collected by a researcher from the Multidisciplinary Academic Division of Comalcalco (DAMC), Universidad Juárez Autónoma de Tabasco (UJAT). This cross-sectional dataset included one sample per participant, collected at varying gestational weeks (4–24), without longitudinal follow-up, and was not generated by the authors of the present study. Sample collection followed the standardized clinical protocol previously described by [4], conducted at the Laboratory of Research in Metabolic and Infectious Diseases (UJAT). Vaginal and cervical samples were obtained by a trained gynecologist using sterile swabs from the ectocervix and posterior vaginal fornix, and stored at 4 °C until genomic DNA extraction. Bacterial detection and quantification were performed using standardized molecular methods applied consistently across all samples. The dataset contains information from pregnant women participating in healthy pregnancy campaigns (August 2018–January 2020) across rural and urban communities in Tabasco, Mexico. It includes sociodemographic data, bacterial presence, Human Papillomavirus, and BV diagnosis.

(b) Data Structure: To understand the structure of the dataset, an exploratory analysis was performed. Table 2 summarizes its main aspects, showing an imbalance between classes, with BV− as the majority class. However, this imbalance does not affect PA methodology, as PA relies on covariance or correlation matrices rather than class proportions [13,18].

The data set comprises three classes of BV diagnosis:

- BV+: Positive BV, indicating presence of the condition.
- Indeterminate: Cases without a clear diagnosis.
- BV−: Normal microbiota.

**Figure 3:**
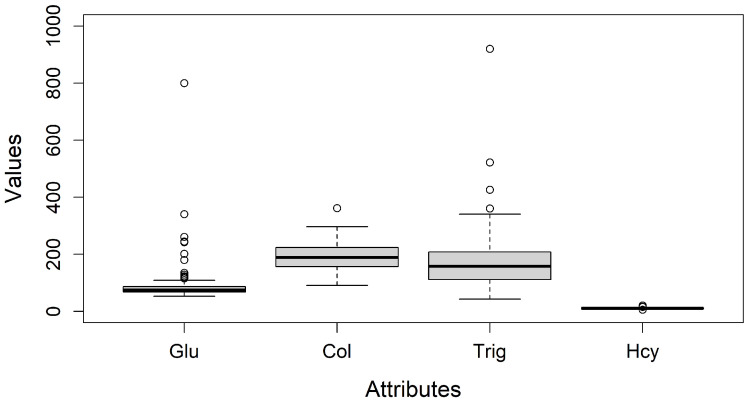
Box and whisker plots of Glu, Col, Trig, and Hcy, showing the distribution and variability of Glucose, Cholesterol, Triglycerides, and Homocysteine.

In addition to class distribution, missing values and outliers were examined as part of the exploratory analysis. Box-and-whisker diagrams were used to identify outliers by visualizing distribution through minimum, first quartile, median, third quartile, and maximum values [29], see Figure 3.

(c) Variable Elimination: Next, consultations were held with the clinical-biological team responsible for data creation to identify variables unrelated to BV. Variables associated with Human Papillomavirus (HPV) were removed, as previous studies found no significant association with BV [4]. This refinement reduced the dataset to 72 attributes and 132 instances, retaining only variables relevant to the study objectives.

(d) Resolving Outliers: To ensure result quality and reliability, missing values and outliers were addressed [30]. A detailed analysis based on boxplot charts, identified outliers in Glucose (Glu), Cholesterol (Col), Triglycerides (Trig), and Homocysteine (Hcy). These attributes could be real values and may indicate additional patient conditions; thus, the clinical-biological expert recommends retaining them for a more comprehensive analysis. Figure 3 presents box plots illustrating their distribution and preserved out-of-range values.

(e) Imputation of Values: Subsequently, missing values were addressed through imputation, replacing null entries to prevent gaps from affecting the analysis. For numerical variables (e.g., age, salary), missing values were substituted with the mean of the attribute, while for categorical variables (e.g., gender, color), the most frequent value (mode) was used [31,32].

In this study, imputation was performed based on the BV diagnosis. For categorical variables, complete instances of each attribute were grouped by diagnosis (BV+, Indeterminate, or BV−), and the mode of each group was assigned to the missing values corresponding to that specific diagnosis. For numerical variables, the mean was applied following a similar process.

Table 3 and Table 4 display the imputed data for the first ten observations of the variables number of pregnancies (N.EMBARAZO) and vaginal discharge (FlujoV). The column DxVBNoMh (Diagnosis of Bacterial Vaginosis without *Mycoplasma hominis*) indicates the BV diagnosis: 1 for BV+ (positive), 2 for Indeterminate, and 3 for BV− (normal microbiota).

The original dataset included 87 variables and 132 instances. After removing irrelevant attributes, 72 variables remained, with the number of instances unchanged. Outliers were identified and retained based on recommendations from the clinical-biological team, who deemed them relevant for interpretation and analysis. Missing values were imputed using the mode for categorical variables and the mean for numerical ones, ensuring completeness and coherence for subsequent analysis.

### 3.2. Application of Path Analysis

**Figure 4:**
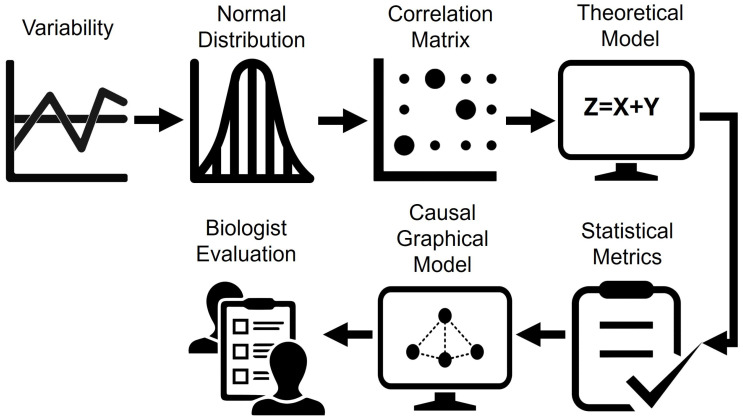
Applied methodology for trajectory analysis: from statistical exploration to evaluation of the causal graphical model.

Figure 4 illustrates the steps of the second phase of the study, which focused on path analysis. This stage included (a) variability assessment, (b) distributional analysis, (c) exploration of the correlation structure, (d) theoretical modeling, (e) validation using statistical metrics, (f) construction of the causal graphical model and (g) review by the expert team.

(a) Variability: Ensuring data variability is crucial for PA validity, as parameter estimation in the path diagram depends on variance and covariances [18,22]. Thus, variance was calculated to confirm that the variables exhibited sufficient variability. Table 5 shows the variance values for some variables in the dataset.

(b) Normal Distribution: After verifying variability, the distribution of the variables was analyzed to determine whether Pearson’s or Spearman’s correlation matrix would be appropriate for assessing correlations. The Q-Q plot and Kolmogorov-Smirnov normality test evaluated adherence to a normal distribution and were applied to each attribute in the dataset, revealing that none exhibited normality.

**Figure 5:**
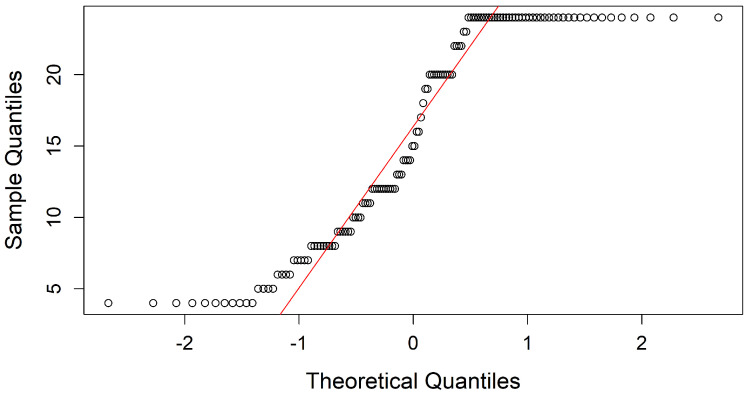
Q-Q plots of SemanasGesta, assessing the distribution of gestational weeks compared to a theoretical normal distribution.

**Figure 6:**
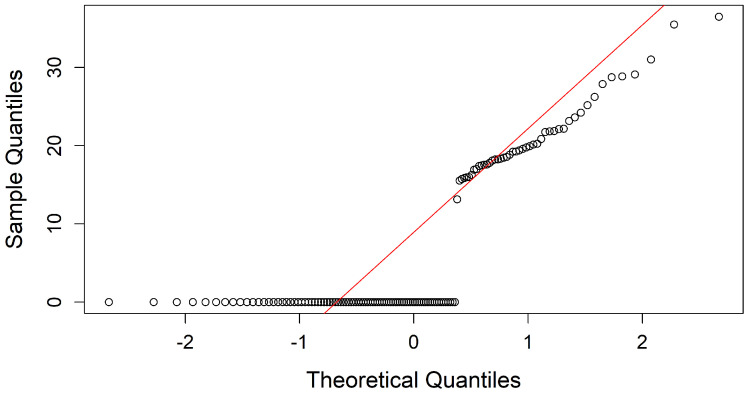
Q-Q plots of Av, assessing the distribution of *Atopobium vaginae* compared to a theoretical normal distribution.

For example, Figure 5 and Figure 6 display the Q-Q plots for weeks of gestation (SemanaGesta) and *Atopobium vaginae* (Av), both of which indicate a non-normal distribution. The test results showed p=0.0001741 with D=0.18819 for SemanaGesta and $p<2.2\times 10^{-16}$ with D=0.40474 for Av, both with *p*-values below 0.05, confirming a deviation from normality.

Different transformation techniques, including logarithmic, square root, reciprocal, and Box-Cox transformations, were applied to improve distribution characteristics. However, due to the nature of the data, no one successfully achieved normality. Table 6 presents the *p*-values of the Kolmogorov-Smirnov test applied to each illustrative variable: weeks of gestation (SemanasGesta), *Mycoplasma hominis* (Mh), *Atopobium vaginae* (Av), *Bacteria Associated with Bacterial Vaginosis Type 2* (BVAB2) and *Lactobacillus crispatus* (Lcrispatus), before and after transformation. N/A values indicate that the transformation was not applicable due to the presence of zeros.

(c) Correlation Matrix: Since the dataset does not follow a normal distribution, the Spearman correlation matrix assessed relationships between variables and their association with BV diagnosis. Five key variables linked to the class attribute (DxVBNoMh) were identified: *Mycoplasma hominis* (Mh), *Atopobium vaginae* (Av), *Gardnerella vaginalis* (Gv), *Megasphaera Type 1* (MT1), and *Bacteria Associated with Bacterial Vaginosis Type 2* (BVAB2) [33].

**Figure 7:**
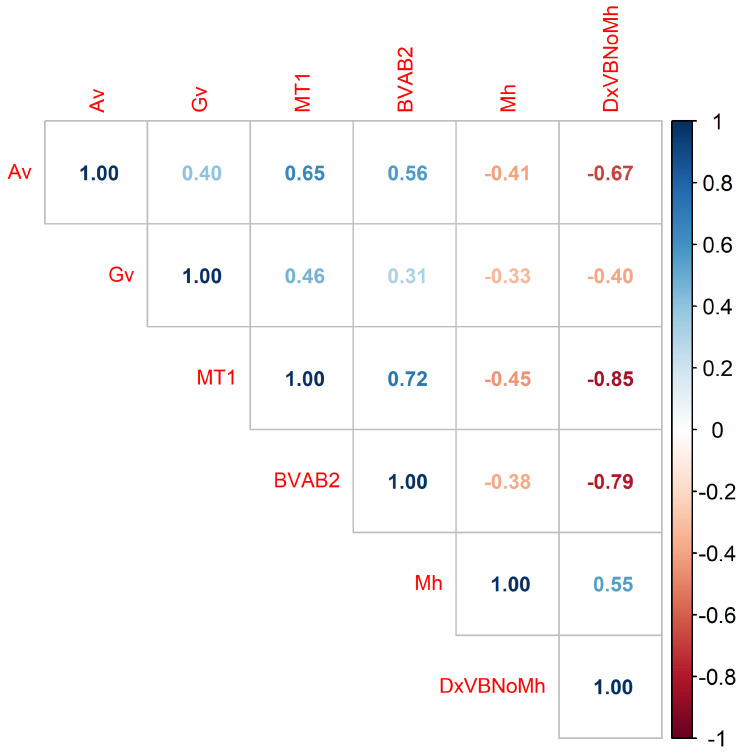
Correlation matrix of DxVBNoMh (BV diagnosis categories: BV+, Indeterminate, BV−) and bacterial presence, highlighting their associations.

**Figure 8:**
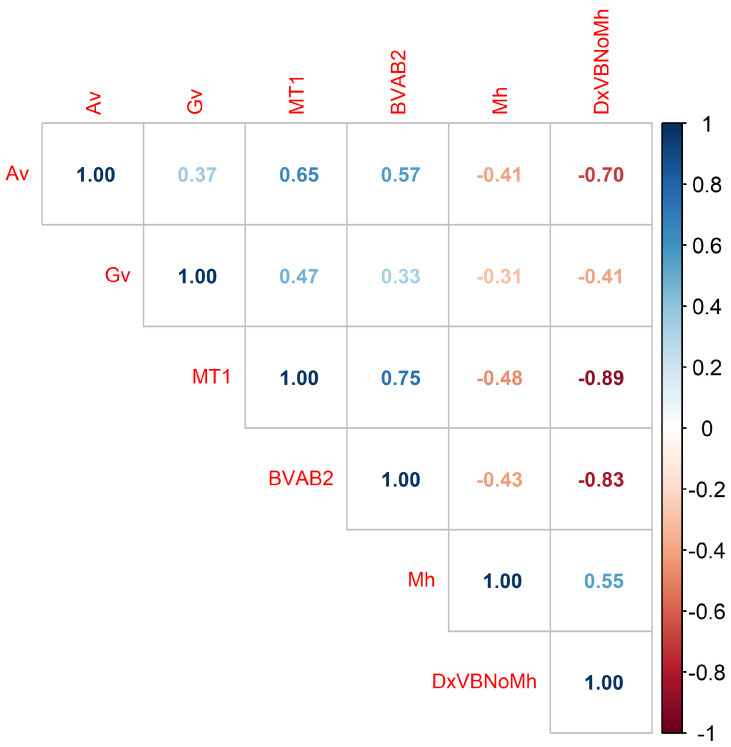
Correlation matrix of DxVBNoMh (BV diagnosis categories: BV+, BV−) and bacterial presence, highlighting their associations.

The correlation matrix presents the coefficients between these variables and the BV diagnosis. Figure 7 includes three diagnostic categories: BV+ (presence of condition), Indeterminate (unclear diagnosis), and BV− (normal microbiota), while Figure 8 considers only BV+ and BV−. Comparing the matrices reveals that the correlation values are highly similar, suggesting that the Indeterminate class has minimal impact and can be omitted without affecting the results.

To investigate the indeterminate class further, future research will conduct an exploratory analysis to determine whether specific patterns justify its inclusion in refined diagnostic models. Thus, Figure 8 is this study’s matrix of primary interest. The last column presents correlation coefficients between the variables and the BV diagnosis: DxVBNoMh with Av (−0.70), Gv (−0.41), MT1 (−0.89), BVAB2 (−0.83), and Mh (0.55). Although some values are low, their clinical relevance lies in their potential to provide essential information for diagnostic and therapeutic decisions [34,35].

(d) Theoretical Model: Based on the correlation matrix, a theoretical model was built as a prerequisite for obtaining the CGM from the dataset. This model is represented by the Equation (7) which specifies the structure of the theoretical model used to construct the CGM. The symbol ∼ denotes a directional relationship, where the variable on the left is modeled as a function of those on the right. The first line indicates that the BV diagnosis without *Mycoplasma hominis* (DxVBNoMh) is predicted using five bacterial variables: Mh, Av, Gv, MT1, and BVAB2. The subsequent lines model MT1 as a dependent variable influenced by each of the other bacteria individually, reflecting its mediating role in the causal pathway. This structure suggests that MT1 may act as a conduit through which other bacteria exert influence on BV diagnosis.

(7) $$\begin{array}{cc} \mathrm{ModC} & \leftarrow \\ ‘\mathrm{DxVBNoMh} & ∼\mathrm{Mh}+\mathrm{Av}+\mathrm{Gv}+\mathrm{MT}1+\mathrm{BVAB}2\\ \mathrm{MT}1 & ∼\mathrm{Mh}\\ \mathrm{MT}1 & ∼\mathrm{Av}\\ \mathrm{MT}1 & ∼\mathrm{Gv}\\ \mathrm{MT}1 & ∼\mathrm{BVAB}2’ \end{array}$$

**Figure 9:**
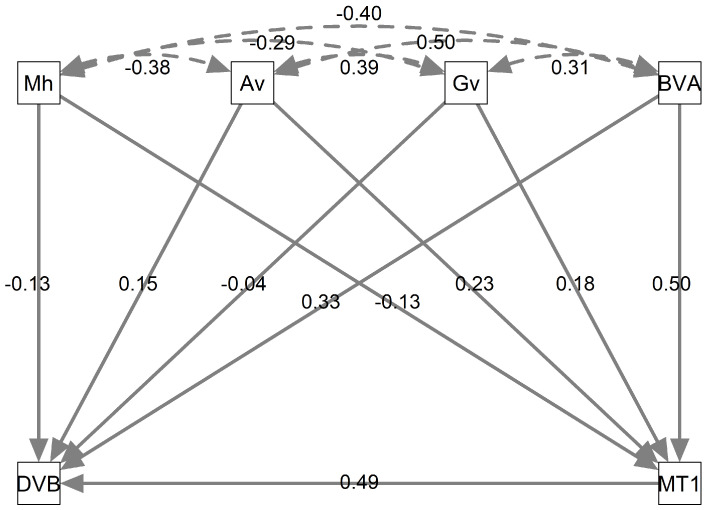
Causal graphical model illustrating the relationships between predictive bacterial attributes and BV diagnosis.

(e) Statistical Metrics: After obtaining the theoretical model, its fit must be evaluated. Table 7 presents the values derived from applying the statistical metrics in Table 1 to Equation (7).

(f) Causal Graphical Model: Based on the theoretical model in Equation (7) and statistical metrics in Table 7, the CGM (Figure 9) was constructed to visually represent causal and indirect relationships among the investigated variables and their impact on DVB. Due to R programming simplifications, some variables were abbreviated: DxVBNoMh as DVB and BVAB2 as BVA. The CGM illustrates causal and indirect relationships (solid lines) between all variables and DVB, as well as covariations (dashed lines). Path coefficients indicate association strength, with values near ±1 representing strong associations and values close to 0 indicating weak associations.

The strongest causal relationship observed was between MT1 and DVB, with a coefficient of 0.49, suggesting that an increase in MT1 level is associated with a higher probability of DVB diagnosis. The weakest causal relationship was between Gv and DVB, with a coefficient of −0.04, indicating a weak negative association. Additionally, indirect relationships were identified, such as the connection between BVAB2 and DVB through MT1, where MT1 acts as an intermediate variable, with coefficients of 0.50 and 0.49, respectively.

Total effects were calculated by summing direct and indirect effects using standardized path coefficients [36,37]. The direct effect corresponds to the path coefficient linking one variable to another, representing their immediate relationship. Indirect effects were determined by multiplying coefficients along causal pathways. Table 8 summarizes the total effects of predictor variables on BV diagnosis.

An interpretation of Table 8 is provided below:

- Mh (−0.1937): Slightly reduces the probability of DVB, suggesting a modest protective effect.
- Av (0.2627): Positively associated with DVB, indicating a higher likelihood of diagnosis.
- Gv (0.0482): Weak positive correlation; a small increase in Gv slightly raises diagnosis probability.
- MT1 (0.4900): Strong positive impact, significantly increasing DVB likelihood.
- BVAB2 (0.5750): Highest positive correlation, indicating a strong association with DVB risk.

(g) Biologist Evaluation: Finally, the CGM was presented to the clinical-biological expert for interpretation, validating the identified causal relationships and assessing their relevance to BV diagnosis. The expert evaluated the causal links based on their biological plausibility, clinical coherence, and consistency with the specialized scientific literature.

Path analysis visualizes bacterial relationships through a diagram, illustrating their connection to BV. In this study, the selected bacteria (Av, Gv, MT1, BVAB2, and Mh) align with those most commonly associated with BV, as reported in specialized biologist literature. The diagram highlights direct and indirect interactions, allowing for calculating each bacterium’s total effect on the diagnosis and identifying the most influential one.

## 4. Results

This study analyzed a dataset of pregnant women tested for BV, applying Path Analysis (PA) to identify coexisting bacteria influencing diagnosis. The original dataset contained 87 attributes and 132 instances, categorized into bacterial vaginosis, indeterminate, and normal microbiota. After exploratory analysis, it was refined to 72 attributes, considering only bacterial vaginosis and normal microbiota.

Given the non-normal distribution of the data, a Spearman correlation matrix identified five bacteria significantly associated with BV: *Mycoplasma hominis* (Mh), *Atopobium vaginae* (Av), *Gardnerella vaginalis* (Gv), *Megasphaera Type 1* (MT1), and *Bacteria Associated with Bacterial Vaginosis Type 2* (BVAB2).

Statistical evaluation confirmed the model’s fit (RMSEA = 0.00, SRMR = 0.00, GFI = 1.00, NFI = 1.00, CFI = 1.00), supporting the construction of the Causal Graphical Model (CGM).

Among the identified bacteria, MT1, BVAB2 and Av exhibited the strongest effects on BV diagnosis, with total effect values of 0.4900, 0.5750 and 0.2627, respectively. The remaining bacteria contributed as follows: Mh (−0.1937) and Gv (0.0482), demonstrating unique interactions in the microbiological ecosystem.

Expert validation confirmed the CGM as an effective visual tool for analyzing bacterial interactions and their impact on BV diagnosis, reinforcing the relevance of causal modeling in microbiological research.

## 5. Discussion

The PA methodology identified five bacteria significantly associated with BV diagnosis, with MT1 and BVAB2 showing the most potent effects. While Av exhibited a positive relationship of lesser magnitude, Gv had a weak positive impact, and Mh showed a potential inverse association. Unlike machine learning models that identify bacterial correlations but do not quantify influence, PA enables the assessment of direct and indirect effects, providing deeper insights into BV diagnosis. The identified bacteria (Gv, Av, Mh, and MT1) align with previous findings [6,7,8], reinforcing the validity of the CGM.

The direct relationship between MT1 and BV diagnosis, as identified by the CGM model, is consistent with the high prevalence of MT1 during pregnancy reported by Glascock et al. [38]. These findings suggest that, unlike other BV-associated bacteria, the physiological changes of pregnancy may favor colonization by MT1, increasing the risk of ascending infection of the upper genital tract. In addition, the indirect effect of MT1 on BVAB2 and Av highlights the role of the heterogeneity of microbial communities for BV development, supporting the proposal that MT1 is frequently associated with a vaginal high-diversity microbiome [38]. Gestational age is known to influence vaginal microbiota composition, potentially affecting the interpretation of infection dynamics throughout pregnancy. However, the cross-sectional design of the dataset restricts temporal follow-up. Future longitudinal applications of PA could capture these temporal shifts, providing a more complete understanding of influential bacterial trajectories.

Although the sample size is relatively small (132 real cases), PA relies on correct model specification given using correlation and covariance matrices rather than class balance, ensuring stable coefficient estimates. Future validation with larger datasets will strengthen its external validity and enhance clinical applicability, demonstrating the value of PA in microbiological research.

## 6. Conclusions

This study applied path analysis (PA) to a dataset of pregnant women to identify causal variables associated with the diagnosis of bacterial vaginosis. Before implementing the model, a structured data preprocessing stage was carried out to minimize bias and ensure optimal performance. Missing numerical values were imputed using the mean, and categorical values using the mode.

Outliers were retained based on the recommendation of a clinical-biological expert, as they reflected other conditions present in the patients. Additionally, variables unrelated to bacterial vaginosis such as those associated with Human Papillomavirus (HPV) were excluded based on prior evidence of non-association. These decisions allowed the dataset to be refined and the analysis to focus on relevant attributes.

PA effectively identified causal relationships between five bacterial variables and BV diagnosis. Among them, BVAB2 exhibited the strongest overall contribution (0.5750), followed by MT1 (0.4900), Av (0.2627), Gv (0.0482) and Mh (−0.1937). These findings align with the previous literature and were validated by a clinical-biological expert, confirming the methodology’s reliability.

In addition to identifying significant associations, path analysis enabled the visualization of direct and indirect relationships between bacterial variables and the diagnosis, allowing the estimation of total effects that clarify each bacterium’s contribution to BV. Beyond its analytical advantages, the CGM could offer clinically valuable insights, supporting gynecological assessments and prenatal care, particularly in low-resource settings.

In future work, we propose validating this CGM using larger and more diverse datasets, exploring its applicability in longitudinal and multicentric research to refine diagnostic precision and treatment strategies in clinical settings.

## Figures and Tables

**Table 1:** Statistical metrics to assess model fit in path analysis.

Metric	Description	Expected Value
RMSEA	Root Mean Square Error of Approximation	<0.05
SRMR	Standardized Root Mean Square Residual	<0.05
GFI	Goodness of Fit Index	≥0.95
NFI	Normalized Fit Index	≥0.95
CFI	Comparative Fit Index	≥0.95

**Table 2:** Summary of the data structure.

Aspect	Description
Attributes	87 variables
Instances	132 observations
Attribute Types	41 categorical and 46 numerical
Observations by Class	BV+ (32), Indeterminate (5), and BV− (95)
Missing Data	609 values
Outliers	143 values

**Table 3:** Data imputed with the mean of the attribute N.EMBARAZO.

Instance	Original Value	Imputed Value	DxVBNoMh
1	1	1.000000	3
2	NA	2.500000	1
3	NA	2.571429	3
4	NA	2.571429	3
5	2	2.000000	3
6	NA	2.571429	3
7	1	1.000000	3
8	1	1.000000	3
9	1	1.000000	3
10	3	3.000000	3

**Table 4:** Data imputed with the mode of the attribute FlujoV.

Instance	Original Value	Imputed Value	DxVBNoMh
1	2	2	3
2	NA	2	1
3	NA	2	3
4	NA	2	3
5	1	1	3
6	NA	2	3
7	1	1	3
8	2	2	3
9	2	2	3
10	2	2	3

**Table 5:** Variance of illustrative variables in the dataset.

Attribute	Variance
Age	74.06
Seeks of gestation (SemanasGesta)	55.83
Glucose (Glu)	5431.24
Cholesterol (Col)	2335.83
*Gardnerella vaginalis* (Gv)	116.56
*Atopobium vaginae* (Av)	111.25
*Bacteria Associated with Bacterial Vaginosis Type 2* (BVAB2)	92.58
*Lactobacillus crispatus* (Lcrispatus)	158.32
*Lactobacillus iners* (Liners)	115.07
*Lactobacillus jensenii* (Ljensenii)	143.58

**Table 6:** Kolmogorov-Smirnov (*p*-values) test before and after transformations applied to each illustrative variable.

Attribute	Original	Logarithmic	Square Root	Reciprocal	Box-Cox
SemanasGesta	0.0001741	$7.878\times 10^{-5}$	0.0001983	$6.869\times 10^{-6}$	<2.2 $\times \;10^{-16}$
Mh	<2.2 $\times \;10^{-16}$	<2.2 $\times \;10^{-16}$	<2.2 $\times \;10^{-16}$	<2.2 $\times \;10^{-16}$	<2.2 $\times \;10^{-16}$
Av	<2.2 $\times \;10^{-16}$	<2.2 $\times \;10^{-16}$	<2.2 $\times \;10^{-16}$	N/A	N/A
BVAB2	<2.2 $\times \;10^{-16}$	<2.2 $\times \;10^{-16}$	<2.2 $\times \;10^{-16}$	N/A	N/A
Lcrispatus	$1.583\times 10^{-7}$	$3.553\times 10^{-12}$	$8.09\times 10^{-9}$	N/A	N/A

**Table 7:** Model metric values.

Indicator	Value	Ideal Value	Good Fit
RMSEA	0.00	<0.05	Yes
SRMR	0.00	<0.05	Yes
GFI	1.00	≥0.95	Yes
NFI	1.00	≥0.95	Yes
CFI	1.00	≥0.95	Yes

**Table 8:** Total effects of bacteria with DxVBNoMh (DVB). Based on Figure 9, computed effect is calculated by algebraic sum of direct effect with indirect effect which in turn is obtained by multiplying the coefficents on the arrows.

Attributes	Computed Effect	Total Effects on DVB
Mh	−0.13+(−0.13×0.49)	−0.1937
Av	0.15+(0.23×0.49)	0.2627
Gv	−0.04+(0.18×0.49)	0.0482
MT1	0.49	0.4900
BVAB2	0.33+(0.50×0.49)	0.5750

## Data Availability

The data presented in this study are available on request from the corresponding author due to data privacy restrictions.
